# Deficits in Sense of Body Ownership, Sensory Processing, and Temporal Perception in Schizophrenia Patients With/Without Auditory Verbal Hallucinations

**DOI:** 10.3389/fnins.2022.831714

**Published:** 2022-04-14

**Authors:** Jingqi He, Honghong Ren, Jinguang Li, Min Dong, Lulin Dai, Zhijun Li, Yating Miao, Yunjin Li, Peixuan Tan, Lin Gu, Xiaogang Chen, Jinsong Tang

**Affiliations:** ^1^Department of Psychiatry, National Clinical Research Center for Mental Disorders, The Second Xiangya Hospital of Central South University, Changsha, China; ^2^Affiliated Wuhan Mental Health Center, Tongji Medical College, Huazhong University of Science and Technology, Wuhan, China; ^3^Guangdong Mental Health Center, Guangdong Provincial People’s Hospital, Guangdong Academy of Medical Sciences, Guangzhou, China; ^4^Department of Neurosurgery, Center for Functional Neurosurgery, Ruijin Hospital, Shanghai Jiao Tong University School of Medicine, Shanghai, China; ^5^Department of Pathology, School of Basic Medical Sciences, Central South University, Changsha, China; ^6^Department of Medical Psychology and Behavioral Medicine, School of Public Health, Guangxi Medical University, Nanning, China; ^7^RIKEN Center for Advanced Intelligence Project, Tokyo, Japan; ^8^Research Center for Advanced Science and Technology (RCAST), University of Tokyo, Tokyo, Japan; ^9^Department of Psychiatry, Sir Run-Run Shaw Hospital, School of Medicine, Zhejiang University, Hangzhou, China; ^10^Zigong Mental Health Center, Zigong, China

**Keywords:** rubber hand illusion, auditory verbal hallucinations, body ownership, sensory processing, temporal perception

## Abstract

It has been claimed that individuals with schizophrenia have difficulty in self-recognition and, consequently, are unable to identify the sources of their sensory perceptions or thoughts, resulting in delusions, hallucinations, and unusual experiences of body ownership. The deficits also contribute to the enhanced rubber hand illusion (RHI; a body perception illusion, induced by synchronous visual and tactile stimulation). Evidence based on RHI paradigms is emerging that auditory information can make an impact on the sense of body ownership, which relies on the process of multisensory inputs and integration. Hence, we assumed that auditory verbal hallucinations (AVHs), as an abnormal auditory perception, could be linked with body ownership, and the RHI paradigm could be conducted in patients with AVHs to explore the underlying mechanisms. In this study, we investigated the performance of patients with/without AVHs in the RHI. We administered the RHI paradigm to 80 patients with schizophrenia (47 with AVHs and 33 without AVHs) and 36 healthy controls. We conducted the experiment under two conditions (synchronous and asynchronous) and evaluated the RHI effects by both objective and subjective measures. Both patient groups experienced the RHI more quickly and strongly than HCs. The RHI effects of patients with AVHs were significantly smaller than those of patients without AVHs. Another important finding was that patients with AVHs did not show a reduction in RHI under asynchronous conditions. These results emphasize the disturbances of the sense of body ownership in schizophrenia patients with/without AVHs and the associations with AVHs. Furthermore, it is suggested that patients with AVHs may have multisensory processing dysfunctions and internal timing deficits.

## Introduction

Schizophrenia is a severe mental disorder characterized by thought and perception disturbances. Individuals with schizophrenia usually have difficulties in distinguishing between reality and fantasy, which may cause unusual experiences of reality. Auditory verbal hallucinations (AVHs) are one of the most common presenting symptoms of schizophrenia, and are usually defined as the sensory perceptions of hearing voices that do not exist, occurs in approximately 74% of patients with schizophrenia (Dierks et al., 1999; Choong et al., 2007). Since the mechanisms underlying AVHs are unknown, researchers have proposed multiple theoretical models, such as unstable memories, source monitoring, top-down attention, and hybrid models of hallucinations (Ćurčić-Blake et al., 2017b).

From the view of cognition, most perception disturbances revolve around self-recognition failures, which means that patients tend to mistakenly attribute self-generated behaviors to an external source (Waters et al., 2010; Graham et al., 2014; Klaver and Dijkerman, 2016). Auditory verbal hallucinations are due to attribution errors, where internal mental events are mistaken as sensations originated from the surrounding environment (McGuire et al., 1995). Some researchers propose that AVHs result from an attentional bias toward internally generated information. This theory is supported by neuroimaging findings that suggest patients with AVHs also show deficits in processing exogenously presented sounds (David et al., 1996; Woodruff et al., 1997; Hugdahl et al., 2009; Kompus et al., 2011). Patients with AVHs can show an impaired performance on auditory attention tasks, such as the dichotic listening (DL) paradigm (Hugdahl et al., 2007). A meta-analysis found that activation in the left primary auditory cortex and the right rostral prefrontal cortex increased in the absence of external auditory stimuli but decreased in the presence of external auditory stimuli (Kompus et al., 2011).

In fact, numerous brain regions associated with audition are also involved in processing other sensory information and the integration of different types of sensory information (Caetano and Jousmäki, 2006; Butler et al., 2012; Knöpfel et al., 2019; Kassuba et al., 2020). Meanwhile, there are interactions between audition and other senses in some ways, such as touch and vision. For instance, long-term exposure to auditory noises can increase sensitivity to tactile frequency (Kassuba et al., 2020). By providing visual stimuli consistent with auditory stimuli, the representation of that sound can be strengthened in the auditory cortex (Atilgan et al., 2018). Moreover, multisensory integration effects can also act on some more advanced functions, such as emotional response and perception (Collignon et al., 2008; Pan et al., 2019), speech and language processing (Righi et al., 2018), and self-perception (Noel et al., 2018). According to the research based on the rubber hand illusion (RHI) paradigm, the sense of body ownership also relies on the reception and integration of self and externally generated multisensory information (Grechuta et al., 2021). Previous studies have shown that auditory cues can help to enhance the illusion, which suggests that auditory information plays a role in the framework of body ownership (Radziun and Ehrsson, 2018). In addition, a recent study has revealed that the sense of body ownership can be impacted by distal auditory signals that are independent of the action (Grechuta et al., 2021). We can speculate that, since AVHs involve abnormal auditory perceptions, they may have an impact on the processing of all types of sensory information, including the sense of body ownership. Although prior studies have established that deficits in multisensory integration are closely correlated with schizophrenia, the studies on the direct relationship between AVHs and integration processing are lacking, and most of them focus on audiovisual integration (Surguladze et al., 2001; Kim et al., 2003; Szycik et al., 2009).

The RHI paradigm is a classic and effective experimental method to induce body ownership illusions *via* the visual and tactile stimulations (Graham et al., 2014). The sense of body ownership relies on multisensory integration. The RHI paradigm induces a distorted sense of body ownership in an attempt to mediate conflicting visual and proprioceptive signals by integrating multisensory input to form a coherent representation of the body and the world (Tang et al., 2015; Klaver and Dijkerman, 2016). Simply put, the participants are more likely to report that the rubber hand is their own hand by hiding the real hand and, brushing the two hands synchronously while watching the fake hand, while these feelings can decrease under asynchronous conditions (Tsakiris and Haggard, 2005). In addition, the illusion can be reduced under circumstances where the rubber hand does not match the subject’s visual body image, such as using a wooden block or changing the position of the subject’s hand (Tsakiris and Haggard, 2005; Costantini and Haggard, 2007).

Due to their disturbed sense of self, patients with schizophrenia can experience abnormal body perception, including impairments in body ownership. Therefore, they are more susceptible to enhanced RHI (Thakkar et al., 2011; Zopf et al., 2021). The use of the RHI in patients with schizophrenia was first described by Peled et al. (2000), who found that patients have more profound experiences of the illusion than healthy controls (HCs), and subsequent studies have reported similar results (Peled et al., 2003; Thakkar et al., 2011; Germine et al., 2013). Furthermore, healthy individuals with psychosis-proneness also have more intensive experiences than those without schizotypal personality traits (Germine et al., 2013). A significant correlation between the severity of body-related perceptual symptoms and the intensity of RHI has been found in patients with schizophrenia (Germine et al., 2013; Zopf et al., 2021). In addition, researchers have observed that the sensitivity to detect temporal differences decreases in patients with schizophrenia by comparing the intensity of the illusion between synchronous and asynchronous conditions, which is related to their increased susceptibility to RHI (Zopf et al., 2021).

In view of the prevalence and specificity of AVH in psychosis, studying AVH is likely to provide a valuable perspective that is distinct from that generated by the study of schizophrenia alone. The RHI paradigm is an accessible way to observe an impaired sense of body ownership in individuals with schizophrenia. Unlike previous studies, it is a process of visual-tactile integration, so we can explore the performance of the patients in this special process and its relationship with specific symptoms. Hence, in this study, we used the RHI paradigm to the schizophrenia patients with AVHs and compared their performance with HCs and the patients without AVHs. We aimed to observe the sense of body ownership and visual–tactile integration in schizophrenia patients with AVHs through an easy and intuitive method. We hypothesized that the illusion intensity of patients with AVHs might be greater than that of patients without AVHs and HCs due to the deficits in their sense of self.

## Materials and Methods

### Participants

A total of 80 patients who met the DSM IV-TR criteria for schizophrenia were recruited from among outpatients attending the Second Xiangya Hospital of Central South University, China, from 2018 to 2019. A total of 36 healthy volunteers with no personal or family history of mental illness were recruited using poster advertisements and screened by matching their age, gender, and education years to the patients. All participants were native Chinese speakers and were interviewed by psychiatrists to evaluate their ability to understand and follow simple instructions. The inclusion criteria required that participants were right-handed, aged between 16 and 45, and were able to understand and cooperate with the research procedures. The exclusion criteria for all participants were (1) substance abuse, (2) a history of head trauma resulting in loss of consciousness, or (3) major medical or neurological illness. According to the presence of AVHs, patients were divided into two subgroups. Patients with AVHs were defined as patients who experienced AVHs at least once a day in the past year, while patients without AVHs were those who had never experienced AVHs since the onset of the disease. The subgroup of patients with AVHs contained 47 patients, while the subgroup of patients without AVHs contained 33 patients. Patients in each group were matched for age, gender, education years, medication, and duration of illness. This study was approved by the Second Xiangya Hospital Ethics Committee (No. S006, 2018), and all participants fully understood the research procedures and provided written informed consent.

### The Rubber Hand Illusion Setup

Each participant sat down at the table opposite the experimenter. They placed their right hand in an open-side box to hide it from view. A life-sized rubber hand, wearing a blue latex glove, was placed to the left of the real right hand. The distance between the two hands was 15 cm. To maintain a consistent appearance, participants were required to wear a blue latex glove on their right hand. In addition, their right arm was covered by a long black cloak, which could prevent the participant from observing the location of their real hand. Two rotating brushes, driven by an electric motor (Xinda XD60D94-12Y-505, China), struck the right index finger of both the real and rubber hands at a constant rhythm of approximately 1 Hz. The experiment included two conditions, namely, synchronous and asynchronous. In the synchronous condition, the two brushes moved simultaneously without interval time. In the asynchronous condition, there was a 500-min delay in the brush that struck the real hand, which meant that tactile stimulation would be given to the participants after the visual stimulation of seeing the brush strike the rubber hand. Before the start of the experiment, the rubber hand was hidden from view.

### Experimental Design

According to the condition of visuo-tactile stimulation, the experiment was divided into two blocks, and all participants experienced both blocks. The stimulation order was determined by coin flipping and was completely random and counterbalanced. Each block consisted of a 2-min induction period followed by a 3-min period during which the participants located their right index finger. Two measures were applied to evaluate the intensity of the illusion, namely, proprioceptive drift and subjective experience.

### Proprioceptive Drift

At the beginning of the task, all participants were asked to make a judgment about the baseline position of their right index finger. Then, the participant was allowed to see the rubber hand and the electric motor started. After a 2-min induction period (Tang et al., 2015; Prikken et al., 2019; Zopf et al., 2021), the participant made a further judgment about their index finger every 1 min. Five judgments would be made in total, and each task lasted for 6 min. All judgments were documented using the readings observed in a ruler placed on top of the box. Drift was defined as the change in the perceived location from the baseline measurement, calculated by subtracting the estimates before the illusion from the estimates after the illusion. The average of the four measurements was used in the analysis.

### Subjective Experience

After completing each task, participants were asked to complete a questionnaire in Chinese consisting of nine statements describing the perceptual experiences and sensations, such as “It seemed as if I were feeling the touch of the paintbrush in the location where I saw the rubber hand” and “I felt as if the rubber hand were my hand” (Botvinick and Cohen, 1998). Answers were scored on a scale ranging from 1 (completely disagree) to 5 (completely agree).

### Clinical Evaluation

Clinical evaluation was performed independently by two experienced senior psychiatrists. The severity of illness was assessed by the 30-item Positive and Negative Syndrome Scale (PANSS) (Kay et al., 1987). For the subgroup of patients with AVHs, the 7-term Auditory Hallucinations Rating Scale (AHRS) was conducted to assess the severity of AVH (Hoffman et al., 2003).

### Statistical Analysis

All statistical analyses were performed using SPSS (version 25, IBM Inc., New York, United States). All statistical tests were two-tailed, and the effects were considered significant if *p* < 0.05. Shapiro–Wilk’s tests were used as normality tests. As the data did not fall into a normal distribution, we used non-parametric tests: the Kruskal–Wallis H test was used in the intergroup comparison; Wilcoxon signed-rank test was used in analyses within the same group; and Spearman’s rank correlation coefficient test was used in exploring the relationship between the severity of symptoms and the intensity of the illusion.

## Results

### Participants

The demographic and clinical characteristics of the participants are summarized in Table 1. The study population consisted of 47 patients with AVHs, 33 patients without AVHs, and 36 HCs. No significant differences were found in gender, age, and education among the three groups. Compared to patients without AVHs, patients with AVHs had higher PANSS total and positive scores. However, no group differences were found for the negative or general scores between the two patient groups.

**TABLE 1:** Demographic and clinical characteristics of patients with auditory verbal hallucinations (AVHs), patients with no AVHs and healthy controls.

	AVHs patients (*n* = 47)	Non-AVHs patients (*n* = 33)	HCs (*n* = 36)	Statistic test (df)	*p* value
**Demographic**					
Gender(M/F)	25/22	15/18	19/17	χ^2^(2) = 0.541	0.763
Age (years)	25.9 ± 5.3 (17–38)	27.0 ± 6.3 (18–42)	26.9 ± 5.7 (19–36)	*F*(2,113) = 0.511	0.601
Education(years)	12.6 ± 2.4 (9–17)	13.7 ± 2.7 (9–19)	13.8 ± 2.6 (6–17)	*F*(2,113) = 2.933	0.057
**Clinical**					
Duration of illness (months)	7.9 ± 5.0	6.0 ± 3.8	–	t(78) = 2.196	0.060
Medication (CPZE mg/day)	639.6 ± 273.3	566.7 ± 411.7	–	t(78) = 0.878	0.344
PANSS Total	58.6 ± 13.6	49.7 ± 15.7	–	t(78) = 2.692	0.009*
PANSS Positive	16.2 ± 4.1	10.4 ± 3.9	–	t(78) = 6.576	<0.001*
PANSS Negative	14.9 ± 5.5	13.1 ± 6.1	–	t(78) = 1.130	0.167
PANSS General	27.4 ± 6.6	26.2 ± 7.6	–	t(78) = 0.820	0.443
P3 item	4.8 ± 0.8	–	–	–	–
AHRS Score	25.9 ± 3.8	–	–	–	–

Values are provided as mean ± SD unless otherwise stated. PANSS, Positive and Negative Syndrome Scale; AHRS, Auditory Hallucinations Rating Scale; CPZE, chlorpromazine equivalent dose. *p < 0.05.

### Medication and the Illusion

Several studies show that the RHI might relate to dopaminergic pathways (Albrecht et al., 2011; Ding et al., 2017; Waldmann et al., 2020), so the medication was entered into the correlation analysis with the illusion. We explored their relationship using Spearman’s rank correlation coefficient. Significance level was defined at α = 0.0125 after Bonferroni correction. There was no significant correlation between medication and synchronous proprioceptive drift [ρ(80) = −0.167, *p* = 0.138], asynchronous proprioceptive drift [ρ(80) = −0.087, *p* = 0.441], synchronous subjective score [ρ(80) = 0.121, *p* = 0.286], or asynchronous subjective score [ρ(80) = 0.240, *p* = 0.032].

### Preliminary Analysis: Baseline Accuracy of Hand Position Estimation

To test whether there were differences in localization at baseline status between the patient groups and the HCs, we first calculated the difference between the baseline readings of the experimenter and that of the participants. Then we conducted a Kruskal–Wallis H test, which showed that there were no significant differences among the three groups in either the synchronous condition [H(2) = 5.314, *p* = 0.070] or the asynchronous condition [H(2) = 3.441, *p* = 0.179]. Therefore, we believed that the patients had no difficulty in hand localization prior to the onset of the illusion-inducing stimulation.

### Proprioceptive Drift

**FIGURE 1:**
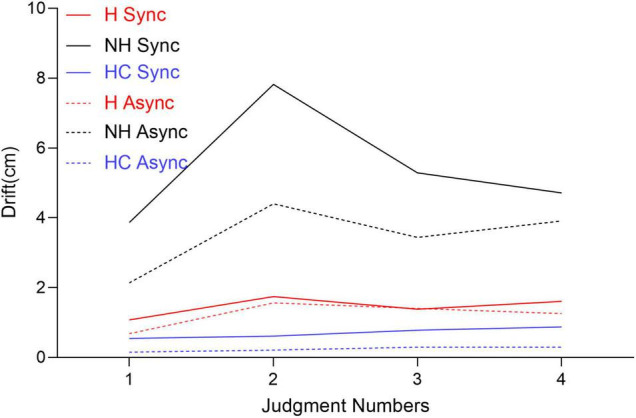
Line graph to show the change in the proprioceptive drift of patients and healthy controls (HCs) over time. Judgments were taken at 1 min intervals.

The perceived hand location varied over time (Figure 1). As shown in the figure, in both patient groups, the maximum proprioceptive drift appeared in the second measurement, while in HCs, it appeared in the last measurement.

**FIGURE 2:**
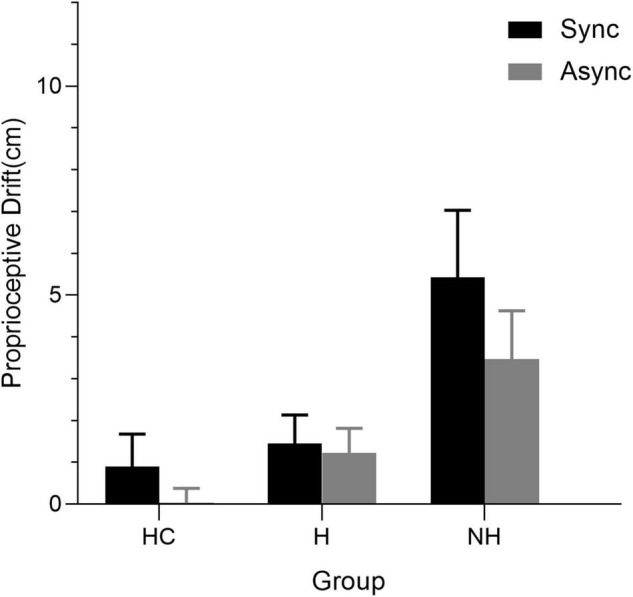
Mean proprioceptive drift of patients with auditory verbal hallucinations (AVHs) (H), patients without AVHs (NH), and HCs under two stimulation conditions: synchronous (Sync) and asynchronous (Async). Error bars reflect 95% confidence intervals.

The Kruskal–Wallis H test showed that whether in the synchronous condition [H(2) = 23.834, *p* < 0.001] or the asynchronous condition [H(2) = 24.198, *p* < 0.001], there was a significant difference in proprioceptive drift among all groups (Figure 2). Pairwise multiple comparisons were performed using Dunn’s pairwise tests with Bonferroni correction, which showed that, under the synchronous conditions, patients without AVHs had the largest mean drift, followed by patients with AVHs and HCs in succession, and the difference in patients without AVHs between patients with AVHs (*p* < 0.001) and HCs (*p* < 0.001) was significant; however, the difference between patients with AVHs and HCs was not significant (*p* = 0.844). Similar results were found in asynchronous conditions, and the pairwise comparisons between the patients without AVHs and the other two groups were significantly different (pH-NH = 0.015, pHC-NH < 0.001, pHC-H = 0.040). The Wilcoxon signed-ranks test showed that the total drift was greater after synchronous rather than asynchronous stimulation in patients without AVHs (*Z* = 1.984, *p* = 0.047) and HCs (*Z* = 2.451, *p* = 0.014), but no significant differences between the two conditions were observed among patients with AVHs (*Z* = 0.321, *p* = 0.748) (Figure 2).

We also performed a generalized linear mixed model (GLMM) to estimate the proprioceptive drift of the patients, and the PANSS score (*p* = 0.515), medication (*p* = 0.743), stimulation condition (*p* < 0.001), and group (*p* < 0.001) were involved in the analysis. The results confirmed that there were no associations between PANSS score or medication and proprioceptive drift.

### Subjective Experience

**FIGURE 3:**
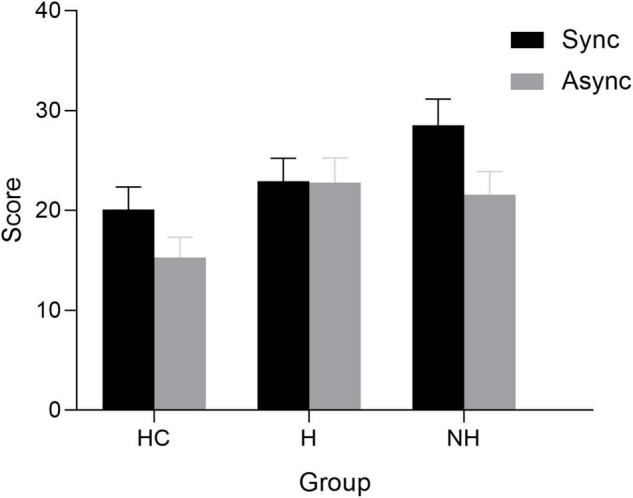
Mean overall subjective experience score of patients with AVHs (H), patients without AVHs (NH), and healthy controls (HC) under two stimulation conditions: synchronous (Sync) and asynchronous (Async). Error bars reflect 95% confidence intervals.

The illusion scores were compared between the three groups using the Kruskal–Wallis H test followed by the *post hoc* Dunn’s test with Bonferroni correction. The results showed that the illusion score was significantly different among the three groups in the two conditions [synchronous condition: H(2) = 19.159, *p* < 0.001; asynchronous condition: H(2) = 19.446, *p* < 0.001] (Figure 3). Under the synchronous conditions, consistent with the proprioceptive drift, the score of patients without AVHs was significantly higher than that of patients with AVHs (*p* = 0.009) and HCs (p < 0.001), while there was no significant difference between the other two groups (*p* = 0.303). Under the asynchronous conditions, each patient group had a higher score than HCs (pHC-NH = 0.002, pHC-H < 0.001), but no significant differences were found between the two patient groups (pH-NH = 1.000) (Figure 3). The synchronous illusion scores were also higher than the asynchronous illusion scores in HCs (*Z* = 4.054, *p* < 0.001) and patients without AVHs (*Z* = 4.276, *p* < 0.001), while no significant difference between the illusion scores under the two conditions was found among patients with AVHs (*Z* = 0.138, *p* = 0.890). Similarly, the results of GLMM showed that medication (*p* = 0.091) and PANSS score (*p* = 0.523) were irrelevant to the illusion score.

### Relationship Between the Illusion and the Severity of Symptoms

We explored the relationship between the illusion intensity and the severity of symptoms using Spearman’s rank correlation coefficient. Both proprioceptive drift and subjective experience under all conditions were entered in the correlation analysis. There was no significant correlation between the measures of the illusion for the two conditions and PANSS total scores or each subscale score in the two patient groups. In addition, for the subgroup of patients with AVHs, the total AHRS score and each item score were included in further analysis. However, no significant correlation was found either.

## Discussion

The aim of this study was to assess the deficits in sense of body ownership and multisensory integration in schizophrenia patients with/without AVHs. We used the RHI paradigm in HCs and patients with/without AVHs and compared their performances. The main findings were as follows: (1) compared to HCs, the illusion of schizophrenia patients with or without AVHs both showed up more quickly and was stronger. (2) Patients with AVHs experienced a weaker illusion than patients without AVHs. (3) Unlike HCs and patients without AVHs, patients with AVHs did not show a reduction in the RHI illusion under asynchronous conditions.

In agreement with previous studies, this study showed that the proprioceptive drift peak appeared earlier and was higher in both patient groups than in HCs. The strong and quick illusion of individuals with schizophrenia revealed that there was an impairment of body ownership, which is a component of self-awareness (Thakkar et al., 2011). In fact, the sense of body ownership involves two processes: the bottom-up integration of multisensory inputs and the top-down prediction (Grechuta et al., 2019). Although disturbances of body ownership in individuals with schizophrenia are often linked with their deficits in multisensory integration, the role of top-down processes cannot be ignored. Ferri et al. designed a new induction procedure for the RHI in which the anticipation replaces the actual synchronized visuo-tactile stimulation and applied it to patients with schizophrenia (Ferri et al., 2013, 2014). The results showed that the patients with schizophrenia had a weaker illusion than the HCs in this paradigm and revealed that patients might have a predictive mechanism different from healthy individuals (Ferri et al., 2014). There have been RHI studies using an unpredictable delay in the asynchronous condition, and their results confirmed the presence of top-down differences between population groups (Botan et al., 2018, 2021). However, there have not been RHI studies on schizophrenia using unpredictable delays. Hence, further RHI studies on schizophrenia can take advantage of unpredictable delays in the asynchronous condition to better understand the predictive mechanisms of patients with schizophrenia.

It is well accepted that a combination of subjective and objective measures is conducive to the better evaluation of the RHI (Klaver and Dijkerman, 2016). In our study, the two measurements’ results were consistent. Nevertheless, contradictory results have been reported in previous studies, which show dissociation between subjective ratings and drift (Shimada et al., 2009; Rohde et al., 2011; Romano et al., 2015). Additionally, studies on the associations between RHI and hypnotizability have suggested that the RHI processes contain implicit imaginative suggestion effects, which may have an impact on the reliability of so-called objective measures such as proprioceptive drift and skin conductance response (Lush et al., 2020, 2021). Lower self-awareness is related to higher hypnotizability (Cardeña and Terhune, 2019). Notably, higher hypnotizability enhances RHI effects and becomes a potential confounding factor (Lush et al., 2020, 2021). However, lower hypnotizability and enhanced RHI effects can both be observed in patients with schizophrenia (Frischholz et al., 1992). Hence, further discussions on hypnotizability and schizophrenia are needed, and relevant confounders should be identified and removed.

However, contrary to our expectation, patients with AVHs showed weaker illusion intensity than patients without AVHs, except that the two groups of patients had similar subjective experience scores under asynchronous conditions. We suggest that there can be several possible reasons to explain the result. The occurrence of RHI is a result of multisensory inputs and integration, so the reduction of the illusion may not mean a better sense of body ownership for patients with AVHs, but a reduction of the response to external stimulations and of multisensory integration. In fact, the development of AVHs is caused not only by the increased activation of endogenously evoked processing but also by the decreased activation of exogenously evoked processing (Kompus et al., 2011). Previous imaging studies have found that the same areas in the auditory cortex will be activated when an external auditory stimulus is not present; instead, when receiving the stimulus, the area will be deactivated (Binder et al., 1996; Hugdahl et al., 1999). There is an inhibitory dysfunction in the anterior cingulate cortex (ACC) of individuals with hallucinations, which leads to a deficient top-down inhibition of automatic activations. Therefore, the self-generated information can compete with the exogenous stimulations for attentional resources, resulting in a reduced response to the exogenous stimulations (Hunter et al., 2006; Hubl et al., 2007). We assume that competition may occur in the response to multisensory stimuli but is not limited to external auditory stimuli.

The RHI is a response to tactile and visual stimuli; in other words, the reduced illusion may represent a reduced response to external stimuli. In addition, the RHI is based on multisensory integration, while impaired multisensory processing but not unisensory processing by individuals with schizophrenia has been detected in some paradigms (de Gelder et al., 2005; Ross et al., 2007; Germine et al., 2013; Stevenson et al., 2017). Among all of the symptoms of schizophrenia, hallucinations are the most closely associated with multisensory integration, and previous studies have shown that the more types of hallucinations the patients experienced, the more severe their impairments were (Williams et al., 2010; Postmes et al., 2014).

A relationship between the audiovisual speech-perception network and hallucinations has been recognized, and this network is involved in the audiovisual integration. In addition, some regions in the impaired auditory association cortex, such as the superior temporal gyrus (STG) and the prefrontal cortex, of patients with AVHs may also play a role in tactile perception (Foxe et al., 2002; Schürmann et al., 2006; Bolognini et al., 2010; Spitzer and Blankenburg, 2012; Vergara et al., 2016; Ćurčić-Blake et al., 2017a; Hjelmervik et al., 2020). Existing studies have shown that external auditory signals can enhance the sensitivity to detect tactile frequency (Crommett et al., 2017). We deduced that the tactile frequency sensitivity in patients with AVHs might be reduced, which further caused an increased sensory asynchrony between tactile and visual stimuli and reduced the illusions. In short, there may be some remarkable overlaps between the regions involved in hallucinations and multisensory integration (Surguladze et al., 2001; Kim et al., 2003; Szycik et al., 2009).

Accordingly, a deficit in visual-tactile integration may also exist in patients with AVHs, contributing to the reduced illusions of patients with AVHs. Another likely explanation is that hallucinations and delusions are two of the most common symptoms of schizophrenia, and delusions may be more prevalent in the group of patients without AVHs. The RHI can be seen as a false belief that the fake hand is one’s own hand, similar to a delusion in a way, and previous studies have confirmed that the RHI effects are positively correlated with delusions in patients with schizophrenia (Germine et al., 2013; Crespi and Dinsdale, 2019; Prikken et al., 2019). It has been found that the experiences of delusions are associated with the structural changes within the insula, and dysfunctions in the region are critical to the increased RHI effects (Cascella et al., 2011; Crespi and Dinsdale, 2019).

An unexpected finding was that there was no difference in the illusion effects of patients with AVHs between the synchronous and asynchronous conditions. In the previous RHI studies on patients with schizophrenia, this phenomenon tended to be associated with their timing deficits (Zopf et al., 2021). It is commonly known that individuals with schizophrenia have a reduced sensitivity to asynchrony, and they usually need longer temporal intervals between stimulations to detect the asynchrony (Foucher et al., 2007; Arzy et al., 2011; Stevenson et al., 2017). This reduced sensitivity has been linked to body-related perceptual symptoms and passivity symptoms (Graham et al., 2014; Zopf et al., 2021). The result of a study using the RHI paradigm was in line with the findings, and it also indicated that the reduced sensitivity was irrelevant to the increased RHI susceptibility (Zopf et al., 2021). In this study, we set the delay time to 500 min, which was not long enough to avoid the normal reduction in RHI typically seen under asynchronous conditions (Graham et al., 2014). We found it in patients without AVHs but not in patients with AVHs, indirectly suggesting that the impairments in temporal processing in patients with AVHs were more severe than in patients without AVHs. Even so, the illusion effect did not become stronger along with it, which confirms the finding mentioned above (Zopf et al., 2021).

These findings are supported by relevant neuroimaging studies, showing that the functional and anatomical changes in the posterior temporal cortex not only participate in the formation of hallucinations (Barta et al., 1990; Levitan et al., 1999; Kim et al., 2003; Cachia et al., 2008), but are also involved in multisensory integration processes, including temporal processing (Miller and D’Esposito, 2005; Stevenson et al., 2011). Some researchers have proposed that there might not be specific brain networks for temporal perception, and instead they widely exist in each neural network (Stevenson et al., 2017). However, most neuroimaging studies on the relationship between multisensory integration, temporal processing in it and hallucinations are focused on audiovisual coupling, and the precise mechanisms and the regions involved with visual-tactile integration in patients suffering from hallucinations are still unknown. Hence, further studies should take more types of sensory integration into account.

## Conclusion

In conclusion, there was a disturbance in the sense of body ownership in individuals with schizophrenia whether they experienced AVHs or not. In addition, those patients with AVHs seemed to show a reduced multisensory processing and integration. Meanwhile, deficits in temporal perception, which indicate impaired multisensory integration, were observed. The reason might be that there are some overlaps between the brain regions involved in multisensory integration and hallucinations. Notably, our findings of temporal perception deficits were in accordance with the lack of facilitation of RHI for the synchronous condition, so measures of time perception should be considered in future studies. The current findings were based on behavioral measures only, whereas past neurobiological studies about the integration process were mostly limited to audiovisual stimulation. Therefore, some neuroimaging tools, such as magnetic resonance or electroencephalogram, can be used in future studies to more deeply explore the underlying mechanisms.

## Data Availability Statement

The raw data supporting the conclusions of this article will be made available by the authors, without undue reservation.

## Ethics Statement

The studies involving human participants were reviewed and approved by the Second Xiangya Hospital Ethics Committee. The patients/participants provided their written informed consent to participate in this study.

## Author Contributions

JT and XC designed the study. JH, HR, JL, MD, and LD collected the samples and clinical information. JH analyzed and discussed the experimental result. JH and HR wrote the first draft of the manuscript. ZL, YM, YL, and PT performed manuscript revision. LG, JT, and XC reviewed the draft and contributed to manuscript preparation. All authors contributed to and approved the final manuscript.

## Conflict of Interest

The authors declare that the research was conducted in the absence of any commercial or financial relationships that could be construed as a potential conflict of interest.

## Publisher’s Note

All claims expressed in this article are solely those of the authors and do not necessarily represent those of their affiliated organizations, or those of the publisher, the editors and the reviewers. Any product that may be evaluated in this article, or claim that may be made by its manufacturer, is not guaranteed or endorsed by the publisher.
